# Type 3 Innate Lymphoid Cells as Regulators of the Host-Pathogen Interaction

**DOI:** 10.3389/fimmu.2021.748851

**Published:** 2021-09-29

**Authors:** Ana Valle-Noguera, Anne Ochoa-Ramos, Maria José Gomez-Sánchez, Aranzazu Cruz-Adalia

**Affiliations:** Department of Immunology, School of Medicine, Universidad Complutense de Madrid; 12 de Octubre Health Research Institute (imas12), Madrid, Spain

**Keywords:** type 3 innate lymphoid cells, infection, pathogens, mucosa, host-pathogen interaction, IL-22, IL-17, ILC3s

## Abstract

Type 3 Innate lymphoid cells (ILC3s) have been described as tissue-resident cells and characterized throughout the body, especially in mucosal sites and classical first barrier organs such as skin, gut and lungs, among others. A significant part of the research has focused on their role in combating pathogens, mainly extracellular pathogens, with the gut as the principal organ. However, some recent discoveries in the field have unveiled their activity in other organs, combating intracellular pathogens and as part of the response to viruses. In this review we have compiled the latest studies on the role of ILC3s and the molecular mechanisms involved in defending against different microbes at the mucosal surface, most of these studies have made use of conditional transgenic mice. The present review therefore attempts to provide an overview of the function of ILC3s in infections throughout the body, focusing on their specific activity in different organs.

## Introduction

Innate lymphoid cells (ILCs) are a subset of lymphocytes lacking the rearranged antigen receptors expressed by adaptive cells, and residing in peripheral tissues, particularly at barrier surfaces. It is now recognized that their location in peripheral tissues enables ILCs to respond promptly to tissue perturbation. Indeed, as a result of their location and effector phenotype, ILCs produce cytokines within hours of activation, as opposed to the number of days required for naive adaptive lymphocytes to be primed, expand, differentiate and enter tissues (1).

ILCs are found in both lymphoid and non-lymphoid tissues, although they are particularly abundant at mucosal surfaces as tissue-resident cells. The nomenclature for ILCs proposed in 2013 classified these cells into three groups based on their function, but recently the International Union of Immunological Societies proposed another classification, considering not only function but also development. Therefore, ILCs are divided into 5 groups: Natural Killer cells (NKs), ILC1, ILC2, ILC3 and Lymphoid tissue inducers (LTi) and they can be distinguished by different biomarkers expressed in each subset (2). Despite being identified as non-T, non-B lymphocytes, they can be considered as the innate counterparts of T lymphocytes. Therefore, regarding their function, ILC1s, ILC2s, and ILC3s mirror CD4+ T helper (Th)1, Th2, and Th17 cells, respectively, whereas natural killer (NK) cells mirror CD8+ cytotoxic T cells. Both ILCs and T cells work together to organize the most appropriate immune response to pathogens. ILC1s and Th1 cells react to intracellular pathogens, such as viruses, and to tumors; ILC2s and Th2 cells respond to large extracellular parasites and allergens; and ILC3s and Th17 cells mainly combat extracellular microbes, such as bacteria and fungi (1).

ILC3s express the nuclear factor retinoic acid–related orphan receptor γ t (RORγt) and the aryl hydrocarbon receptor (AhR) both of which are essential for their development and function (3, 4). Two subsets of ILC3s can be distinguished on the basis of the cell surface expression of Natural cytotoxicity receptors (NCR) and have been termed as NCR^+^ILC3s cells (Nkp46^+^ILC3s in mice and NKp44+ ILC3s) or NCR^-^ ILC3s (3, 5). As the innate counterparts of T helper 17 (Th17), ILC3s are the early source of interleukin 17 (IL-17) and IL-22 during infections. They can also produce granulocyte-macrophage colony-stimulating factor GM-CSF, which plays a role in gut tolerance by inducing IL-10 secretion in macrophages and dendritic cells (DCs) that maintain the regulatory T (Treg) population (6). In mice, NKp46^+^ ILC3s are also dependent on T-box transcription factor TBX21 (T-bet) and can also produce interferon-gamma (IFN-γ) (7, 8). Ltis, previously included in ILC3s subset, are also strictly dependent on RORγt. Consequently, in many studies with conditional transgenic mice, which have depleted genes in RORγt-expressing cells, the function of the protein under study might also play a role in Ltis, rather than only in ILC3s. LTi cells were discovered in 1997 and identified as a discrete subset of lymphoid cells that are essential for the development of peripheral lymph nodes (LN) and Peyer’s patches during embryonic life (9). Embryonic LTis, which are involved in embryonic LN formation, are replaced in the adult by LTi cells derived from the bone marrow. In the adult mouse, LTi-like cells are present in high numbers within the adult gut mucosa; they express c-Kit and C-C-C chemokine receptor (CCR)6, but not NCRs. In recent years, some research has revealed that CCR6^+^ILC3s express MHC-II which, through antigen presentation, can induce apoptosis to commensal-specific CD4^+^T cells contributing to maintaining microbiome tolerance (10).

Cytokine release not only constitutes a defence mechanism but also helps maintain homeostasis and the mucosal barrier. IL-22 and IL-17 signaling promotes the production of antimicrobial peptides and regulates the expression of tight junction components in endothelial cells. Therefore, loss of IL-22 production gives rise to dissemination of intestinal bacteria, producing chronic body inflammation and resulting in susceptibility to infections, such as *Citrobacter rodentium* (4, 11, 12). IL-22, produced by ILC3s, is essential in the early phase of infection (11, 13). Furthermore, IL-17 production by ILC3s is also involved in combating fungal pathogens such as *Candida albicans* (14). The molecular mechanisms underlying ILC3 cytokine release for defence against pathogens have been well researched, and in the last few years ILC3s have been found to be regulated by different factors such as diet, the nervous system, circadian rhythms, microbiota and other immune cells (3, 4, 15, 16). Although ILC3 defence against pathogens has mostly been described in the gut, where they combat mostly extracellular bacteria, they also play an important role in other organs like lungs or skin. Therefore, the present paper discusses recent research on the role of ILC3s in infections by different pathogens, summarizing the cell signaling pathways involved in the activation of ILC3s, and analyzing each pathogen-specific tissue.

## Defensive Function of ILC3s in the Skin

### IL-17-Producing ILC3s Together With γδ T Cells Are Essential in the Defence Against the Epidermal Infection of *Staphylococcus aureus*

**Figure 1:**
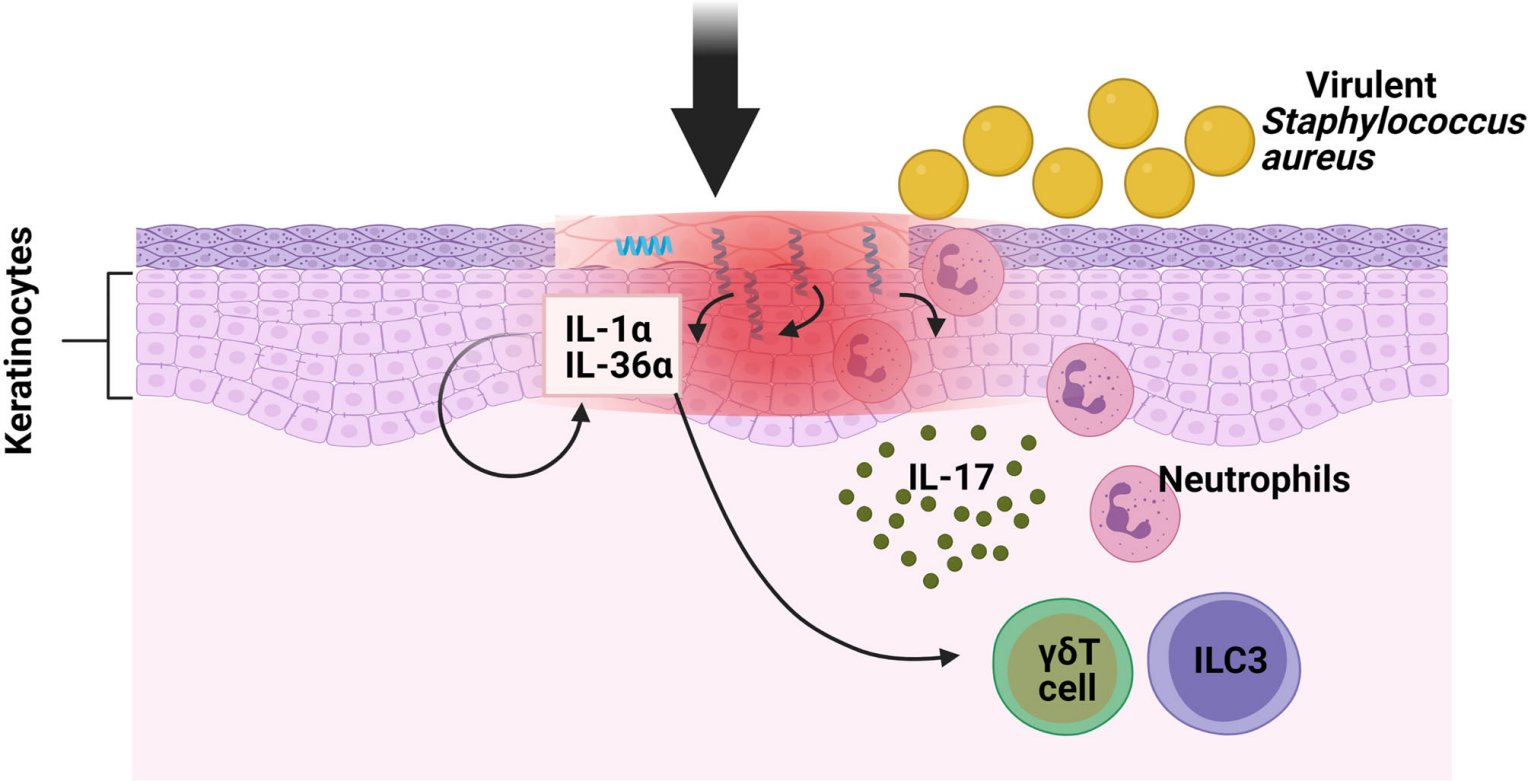
Function of ILC3s against pathogens in oral mucosa. *S. aureus* causes the keratinocytes of the skin to produce IL-1α and IL-36α. These interleukins bind to their receptors in ILC3s and γδ T cells, inducing the production of IL-17, which promotes neutrophil infiltration.

*Staphylococcus aureus*, a gram-positive bacterium, resides in 10–20% of healthy individuals, with the skin surface as its major infection site (17). The cutaneous immune response to *S. aureus* involves both the innate and adaptive immunity. Neutrophils represent the first-responder phagocytic cells that are recruited to the site of infection to help kill pathogens. IL-17A and IL-17F have been shown to promote neutrophil recruitment and previous research found that mice deficient in both cytokines developed spontaneous *S. aureus* skin infections but did not have an increased susceptibility to a systemic *S. aureus* challenge (18). Regarding the role of IL-17 against *S. aureus* cutaneous infections, mice deficient in γδ T cells *(*T cell receptor δ deficient; TCRδ^−/−^) developed larger skin lesions with higher bacterial counts, as well as an impaired neutrophil infiltration and induction of IL-17 compared with WT mice (19). Interestingly, when these TCRδ^−/−^ mice were treated with an anti-CD90 monoclonal antibody (Mab), which depleted their ILCs, their disease scores were seen to be significantly lower in comparison with mice treated with control Mab, thus indicating that both ILCs and γδ T cells contributed to skin inflammation in response to *S. aureus*. Regarding the mechanism involved, the critical role of Myd88 for pathogen colonization was determined by means of *Myd88^−/−^* and *Myd88* ^fl/fl^ K14Cre mice [conditional deletion of Myd88 within keratinocytes (KCs)]. Remarkably, the induction of IL-1α and IL-36α produced by *S. aureus* in *in vitro* primary KCs from *Myd88^−/−^* mice was abolished compared with wild type (WT) cells. This provoked reduced IL-17A-producing cells (ILC3s and γδ T cells) infiltrating into the skin of *Myd88^−/−^* mice following epicutaneous infection. In conclusion, in order to combat the epidermal infection caused by *S.aureus*, there is an induction of IL-17-producing cells (ILC3s and γδ T cells) because of the Myd88-dependent IL-1α and IL-36α production by KCs (**Figure 1**) but further investigations are needed to determine the specific contribution of ILC3s in this model (20).

## Role of ILC3s in Oral Mucosa

### Complementary IL-17 Response by ILC3s and T Cells Is Required to Combat Infection by *Candida albicans*

*C. albicans* is an opportunistic pathogenic fungus considered to be a common member of the human microflora. One of the tissues most frequently affected by *C*. *albicans* is the oral cavity (21). Previous data had proposed ILCs as the IL-17A secretory cells at the onset of oropharyngeal candidiasis (OPC) in *C. albicans-*infected mice, sufficing for protection in *Rag1* ^−/−^ mice but not in *Rag1* ^−/−^ mice treated with anti-CD90 Mab. Indeed, IL-17A mRNA expression in the tongue at 24 h post-infection did not differ between the WT mice and the mice lacking the MHC-II, γδ T cells or NKT cells; however it did decrease in the in IL-23–deficient mice, suggesting that the fungal control was due to ILC function (14). However, the susceptibility in the early response was not analyzed. These results were challenged when mouse models that could not rearrange the TCR were susceptible to OPC, a fact that highlights the importance of T cells in the defence against *C. albicans.* Nonetheless, both of these studies analyzed indirect IL-17 production by antibodies. This has recently been demonstrated ﻿by direct visualization of IL-17A and IL-17F cytokines ﻿in the infected tongue of three IL-17-producing cell types: ﻿αβ T cells, γδ T cells and ILC3s (22) with the use of reporter transgenic mice. Interestingly, selective lack of nTh17 (unprimed population of CD4^+^ T cells) or γδ T cells did not affect fungal control (23), but elimination of both T cells and ILC3s was seen to be necessary to produce the high degree of susceptibility to OPC as with the IL-17RA or IL-17RC-deficient mice. Finally, the mechanism proposed for the production of innate IL-17 in the oral mucosa was through the induction of IL-1β, IL-6 and IL-23 by Langerin+ DCs, which are ﻿tissue-resident ﻿mononuclear phagocytes (MNPs) (22). To conclude, the coexistence of these three different but complementary cell types (nTh17, ILC3s and γδ T cells) is essential for﻿ the IL-17 response to the fungus in oral mucosa.

## Function of the Tissue-Resident ILC3s in Liver

### ILC3s Play a Critical Role in Liver Fibrosis

A wide range of viruses can cause acute or chronic hepatitis and liver infections, for example, Adenovirus (Ad) or lymphocytic choriomeningitis virus (LCMV) which is a prototypical virus found throughout the world and used in animal models of acute and persistent hepatitis (24). It has been demonstrated that the source of the early IL-17A/F production in liver after Ad and LCMV infection in mice was γδ T cells and ILCs, mostly the NKp46^−^ ILC3 population. Importantly, IL-17 source was mostly from ILC3s because Ad-induced hepatitis in ﻿γδ^−/−^ (TCRδ^−/−^) mice did not affect early IL-17A production or Th1/cytotoxic T-lymphocyte responses (25). However, there is a need to use knockout (KO) mice depleting ROR*γ*t^+^ cells or NKp46^+^ cells in this model in order to confirm that specifically IL-17-producing ILC3s are critical for the defence against adenovirus infection.

**Figure 2:**
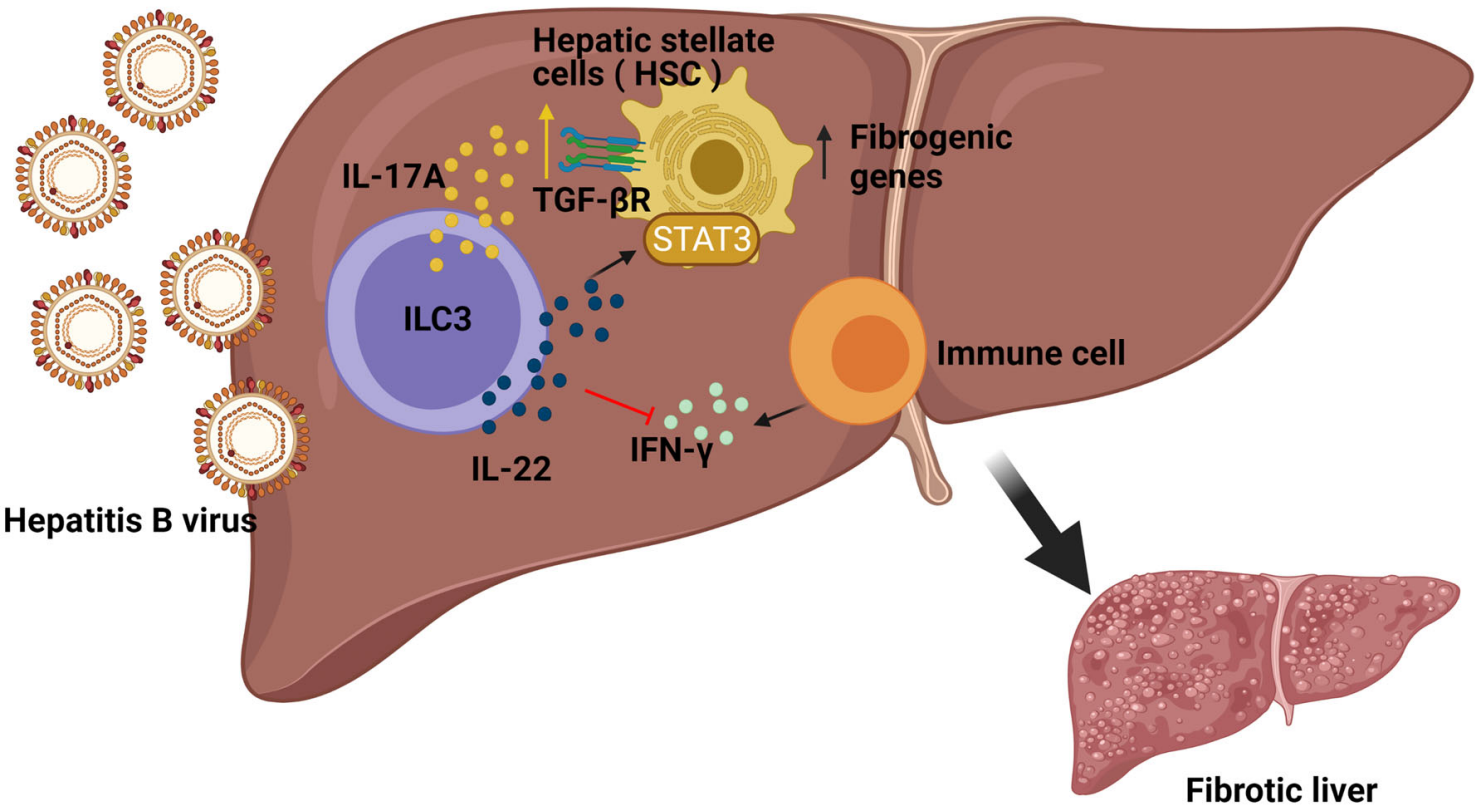
Role of ILC3s in the development of fibrosis induced by Hepatitis B (HBV) infection. In HBV infection, ILC3s can directly promote the expression of fibrogenic genes in hepatic stellate cells (HSC) in non-contact manners by producing IL-17A and IL-22. Additionally, ILC3s also have indirect fibrogenic effects by producing IL-22 to suppress interferon IFN-γ (an anti-fibrotic cytokine) production by other immune cells.

Hepatitis can develop into fibrosis and subsequently to cirrhosis, the latter being responsible for liver morbidity and mortality. In this regard, it has previously been demonstrated that ILC3s can contact directly with the hepatic stellate cells (HSCs) (26), which might affect the progression of liver fibrosis. The frequencies of ILC3s, as well as their production of IL-17A and IL-22, were considerably higher in chronic hepatitis B (CHB) and Hepatitis B Virus (HBV)-related liver cirrhosis (LC) patient subgroups compared to the healthy control group (HC), correlating to a more severe phenotype. In order to study the mechanism at play, pre-stimulated sorted ILC3s were co-cultured with LX-2 cells (human HSC line) revealing a greater proliferation and a higher expression of fibrogenic genes in the LX-2 cells, both in direct and indirect contact, suggesting the critical role of secreted cytokines rather than direct cell contact. Indeed, neutralizing either IL-17A or IL-22 in the cell culture reverted the results, thus revealing a regulation of the expression of the TGF-β receptor and a STAT3 activation in the HSC cell line. In addition, *in vitro* experiments demonstrated that ILC3s had also an indirect mechanism towards fibrosis by producing IL-22 which in turn suppressed IFN-γ (a well-known anti-fibrotic cytokine) production by other immune cells. Finally, to determine the *in vivo* effects of ILC3s in liver fibrogenesis, the carbon tetrachloride (CCl4) murine model was studied in *Rag1* ^−/−^ mice treated with anti-CD90.2 Mab, showing significantly lower HSC activation and less accumulation of the extracellular matrix (ECM) in liver compared with the non-ILC-depleted mice. Remarkably, adoptive transfer of ILC3s from WT to ILC-depleted *Rag1* ^−/−^ mice augmented HSC activation and ECM accumulation in liver, thus providing the *in vivo* pro-fibrotic effects of ILC3s in CCl4-induced mouse liver fibrosis (27) (**Figure 2**). In summary ILC3-produced cytokines influence the HSC cells, which contributes to liver fibrosis.

## Innate Protection of ILC3s in Lung

ILC3s provide innate protection against pathogens within the lung, specifically against different primary or opportunistic pathogens (28) producing IL-17 and IL-22 upon stimulation (29).

### IL-22 Producing Cells, Including ILC3s, Minimize Lung Inflammation During Influenza A Virus and Protect Against Secondary Bacterial Infections

The influenza viruses constitute some of the most predominant human respiratory pathogens, causing substantial seasonal and pandemic morbidity and mortality. ILC3s may play a role in the immune response to pulmonary viral infection due to the importance of the secretion of IL-22 and IL-17. Although IL-17-deficient mice present less lung injury following a viral infection (30). IL-17 has been shown to help to prevent secondary bacterial infections. Co-infection experiments with influenza and *S. aureus* showed that influenza triggered IFN-β production, which inhibited IL-17 production by T cells (31) and suppressed the nuclear factor kappa-light-chain-enhancer of activated B cells (NF-kβ) activation, thus increasing the chances of contracting a secondary pneumonia with *S. aureus* (32). However, there is a need for further research to establish the function of ILC3s, due to the fact that the role of only Th17 was demonstrated in this model. As for IL-22, a murine model of influenza A (IAV) viral infection followed by a secondary *Streptococcus pneumoniae* bacterial infection provoked an increase in RORγt^+^ cells and IL-22^+^ ILC3s in the lung. In addition, while IL-22-deficient mice exhibited no change in viral clearance, their survival was seen to be severely impaired after *S. pneumoniae* secondary infection (33). Indeed, a more recent study showed that infected transgenic IL-22 binding protein KO mice with influenza followed by *S. aureus* or *S. pneumoniae* infection exhibited greater bacterial clearance, a lower mortality rate from secondary bacterial infection, and enhanced airway epithelial integrity (34). In summary, IL-22 has been shown to reduce lung damage following an infection by influenza A infection and to protect against secondary bacterial infections. However, the specific contribution by ILC3s in this model should be demonstrated depleting CD90^+^ or ROR*γ*t^+^ cells in *Rag^−/−^* mice or NKp46^+^ cells.

### ILC3s Produce IL-22 After Acute *Aspergillus fumigatus* Exposure

**Figure 3:**
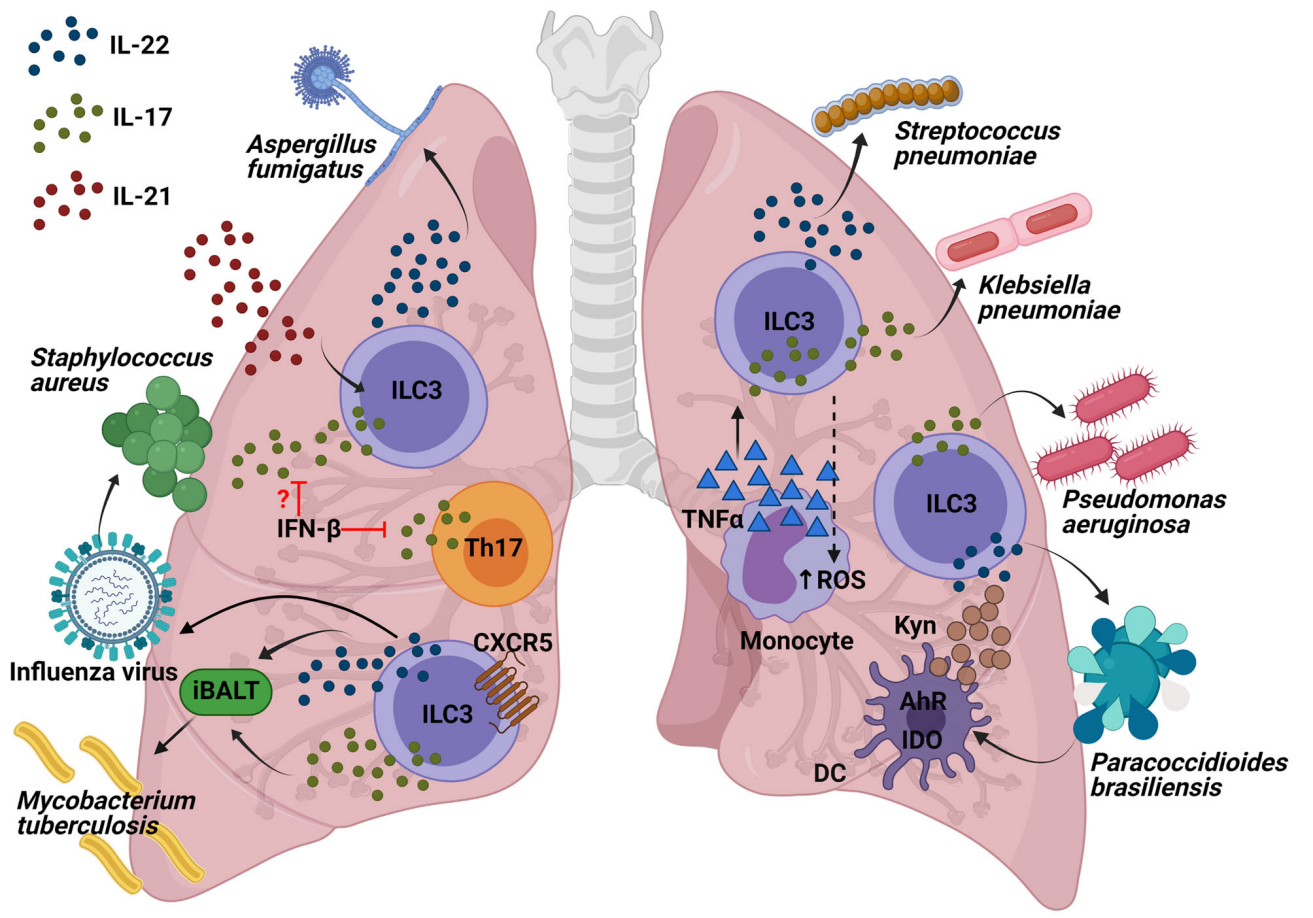
Interleukins produced by ILC3s and the molecular mechanisms of their role to protect against lung pathogens. Production of IL-17 and IL-22 by ILC3s following influenza infection might decrease the susceptibility to *Staphylococcus* infections. Innate-like lymphocytes are involved in the production of IL-22 following *A. fumigatus* exposure, being IL-21 the interleukin required for the maintaining of optimal numbers of iNKT cells and ILC3s. Both IL-17 and IL-22 from ILC3s are involved in the formation of iBALT which helps fight against *M. tuberculosis*. Recruitment of IL-22-producing ILC3s to the lung is required for protection from *S. pneumoniae* infection. IL-17 production in *P. aeruginosa* infection might come from ILC3s and it is essential in the defense against this pathogen. The recruitment of monocytes producing TNF-α increases IL-17-secreting ILCs which has been demonstrated to be important in the clearance of *K. pneumoniae*. AhR is important for IDO expression within the dendritic cells (DCs) as well as their production of kynurenines (Kyn). One of Kyn’s roles is to promote the IL-22 and IL-17-producing ILC3 function in order to fight against *P. brasiliensis*.

*A. fumigatus* is an environmental filamentous fungus which threatens the life of immunocompromised individuals, causing invasive aspergillosis and allergic disease (35). Genetic deficiency in or neutralization of IL-22 results in impaired clearance of *A. fumigatus*, indicating the critical role of IL-22 in pathogen elimination during acute infection (36); neutralization of IL-22 also improves lung function after chronic fungal exposure illustrating that IL-22 drives lung inflammatory responses that have a negative impact on lung function (37). Interestingly, it has been observed that innate and innate-like lymphocytes were involved in the production of IL-22 following 48h of *A. fumigatus* exposure in *Il-22* Cre *R26R* ^eYFP^ reporter mice, in which yellow fluorescent protein (YFP) expression marks IL-22-producing cells. Moreover, lung IL-22 production was completely dependent on IL-7, partially dependent on IL-21, and negatively regulated by IL-15. Employing deficient mice for these interleukins, it was observed that IL-7 appeared to be essential with regard to maintaining invariant natural killer T (iNKT) cells and ﻿γδ T cells in the lung after fungal exposure; IL-21 for maintaining optimal numbers of iNKT cells and ILC3s and as a negative regulator of ﻿γδT cells; moreover, IL-15R signaling did not affect any of the absolute numbers of innate cells but iNKT cells and ﻿γδ T cells displayed higher levels of intracellular IL-22 in deficient mice of IL-15R but not in ILC3s (38) (**Figure 3**). In conclusion, the exposure to *A. fumigatus* can induce IL-22 in ILC3s and innate-like lymphocytes, which could drive lung inflammation; however, this should be demonstrated in mouse models depleting specifically ROR*γ*t^+^ cells, iNKT or ﻿γδ T cells in order to show the contribution of each population.

### ILC3s Regulate the *Mycobacterium tuberculosis* Burden During Infection Through the Formation of Inducible Bronchus-Associated Lymphoid Tissue in Lungs

*M. tuberculosis* (Mtb) is an aerobic bacillus belonging to the Mtb complex, which constitutes the group of mycobacterial pathogens that cause tuberculosis in mammalian species (39). A recently published article has highlighted for the first time the importance of ILC3s in the control of Mtb infection. Ardain et al. demonstrated that ILC3s accumulated rapidly in the lungs of Mtb-infected mice, coinciding with alveolar macrophage accumulation, which has been confirmed in humans through examination of lung tissue from Mtb patients. Importantly, mice lacking ILCs (*Rag2^−/−^ cγc^−/−^* mice) exhibited less early alveolar macrophage accumulation and a lower level of Mtb than the control mice. Notably, an increased load of early Mtb in *Rag2^−/−^ cγc^−/−^* mice could be reverted by adoptive transfer of sorted lung ILCs from *M. tuberculosis*-infected control mice that expressed CCR6, RORγt and AhR. These results were further confirmed in mice with specific depletion of ILC3s (*Ahr* ^fl/fl^ *Rorc* Cre and *Cbfb* ^fl/fl^ Nkp46 Cre mice), which exhibited higher, early and late, Mtb burdens. In order to investigate the possible mechanisms, the migration of mouse ILC3s in response to C-X-C Motif Chemokine Ligand 13 (CXCL13) was confirmed by means of *in vitro* assays. The receptor C-X-C motif chemokine receptor 5 (CXCR5) expressed in ILC3s is important for the formation of inducible bronchus-associated lymphoid tissue (iBALT), the formation of which showed a decrease in ILC3-depleted, *Il-17* ^−/−^ *Il-22* ^−/−^ and IL-23-depleted *M. tuberculosis*-infected mice. However, iBALT formation was not entirely *via* ILC3s, as it was also observed in neonatal *Rorc^−/−^* mice. Similarly, *Cxcr5* ^−/−^ mice also exhibited an increase in lung Mtb colony forming units (CFUs) and decreased accumulation of ILC3s within lymphoid follicles, as well as reduced formation of iBALT structures. All these data support a protective role for ILC3s in regulating early Mtb control through the production of IL-17 and IL-22 with the formation of iBALT structures in a CXCR5-dependent manner (40) (**Figure 3**). Other published studies have confirmed the importance of both NKp46^+^ ILC3s and Ltis in Mtb infection, which were found to have accumulated in the lungs of mice challenged with aerosolized Mtb (41). In short, ILC3s are important not only for the defence against extracellular pathogens, as has previously been shown, they are also essential for controlling infection by the intracellular bacterium *M. tuberculosis*.

### IL-22-Producing ILC3s in the Gut Are Essential to Fight Against *Streptococcus pneumoniae*

*S. pneumoniae* is a gram-positive bacterium that is a common cause of pneumonia, septicaemia, and meningitis. As for IL-22, during *S. pneumoniae* infection, it has been seen to be produced by CCR6^+^ ILC3s in a MyD88 dependent manner and triggered by DCs (42). Interestingly, IL-22 has recently been found to be essential in neonate mice, because recruitment of IL-22-producing ILC3s to the lung, by commensal microbes in the gut, was required for protection from *S. pneumoniae* infection. Treatment of RORγt diphtheria toxin receptor (DTR) newborn mice (*Rorc* Cre mice crossed with *Rosa26*-iDTR mice; *Rorγt* ^iDTR^ *)* with DT diminished the number of ILC3s in the lungs and reduced IL-22 in bronchial lavage fluid (BAL), making them more susceptible to pneumonia. Importantly, it was reversed by transferring lung ILC3s. Indeed, reduced concentrations of IL-22 as well as lower numbers of lung IL-22^+^ ILC3s in BAL were found in human newborns exposed to prolonged durations of antibiotics, thus contributing to increased susceptibility to pneumonia, which was reversed by transfer of commensal bacteria after birth. Intestinal CD103^+^CD11b^+^ DCs were able to capture antigens from commensal bacteria to induce the expression of the lung homing signal CCR4 in ILC3s (43). Later on, transgenic *Rorc* ^GFP/+^ newborn mice were used to confirm the need for pulmonary IL-22-producing ILC3s as a defence against *S. pneumoniae* infection. Furthermore, part of the development of this population in the lungs is generated by ILC precursors expressing the transcription factor promyelocytic leukemia zinc finger (ZBTB16), because their absence reduced ILC3 numbers from birth and throughout adulthood. The expansion and maturation of pulmonary ILC precursors was mediated by the insulin-like growth factor 1 (IGF-1) from alveolar fibroblasts; consequently, co-transplanting the common lymphoid precursors from newborns lacking IGF1R on ZBTB16^+^ ILC precursors in *Rag2^−/−^ cγc^−/−^* mice (lacking all ILC subsets) made them susceptible to *S. pneumoniae* intratracheal challenge (44) (**Figure 3**). More recently, prophylactic intranasal administration of IL-7, an important factor for RORγt^+^ cell survival and homeostasis, has been found to increase the number of RORγt^+^ innate T cells in the lung (NKT, γδT cells and mucosal-associated invariant T (MAIT) cells) and to enhance expression of IL-17A, resulting in a reduction of bacterial burdens upon *S. pneumoniae* challenge (45). In general terms, this brings us to conclude that some of the factors for the generation of pulmonary IL-22-producing ILC3s involve: the commensal microbes in the gut, ZBTB16^+^ ILC precursors, and the expression of IGF-1 by the alveolar fibroblasts. IL-22-producing ILC3s are important both in the newbornhood and adulthood to control a *S. pneumoniae* infection and prevent pneumonia.

### IL-17 and IL-22 From ILC3s Are Essential for Combating Against *Klebsiella pneumoniae* Infection

*K. pneumoniae* is a gram-negative bacterium, found in the normal flora. It can cause different clinical diseases including pneumonia. In recent years, *Klebsiella* species have become relevant pathogens in nosocomial infections (46). A mouse model of *K. pneumoniae* infection was used to describe how the recruitment of monocytes producing TNF-α increased IL-17-secreting ILCs which potentiated reactive oxygen species (ROS) production and, therefore enhanced bacteria killing by monocytes. Indeed, both T cell deficient and WT mice exhibited a similar bacterial clearance, but *Rag2^−/−^cγc^−/−^* mice and *Rag2^−/−^* depleted of ILCs by means of an anti-CD90 MAb led to increased bacterial burden and mortality. Exogenous IL-17 in *K. pneumoniae*-infected *Rag2^−/−^cγc^−/−^* mice were able to clear the pathogen. Remarkably, an adoptive transfer of ILCs from *Rag2* ^−/−^ (but not *Il-17a^−/−^Rag2* ^−/−^) mice reduced the bacterial burden in the lungs of recipient *Rag2^−/−^ cγc^−/−^* mice, thus demonstrating the importance of innate IL-17-producing cells in the defence against *K. pneumoniae* (**Figure 3**). Moreover, IL-22 did not seem to be significant in the early defence against *K. pneumoniae* since its clearance was similar both in *Il-22^−/−^* and in the WT mice (47). However, the lung burdens of infected *Rag2^−/−^ cγc^−/−^* mice were significantly reduced through addition of exogenous IL-22. Indeed, a distinct group of IL-17^+^, IL-22^+^ and inducible T cell costimulatory molecule–positive (ICOS^+^) ILC3s was detected to be essential for host resistance against *K. pneumoniae* in lungs from infected *Rag2^−/−^* mice, using single cell RNA sequencing (48). In conclusion, IL-17 from ILC3s potentiates the bacterial clearance of *K. pneumoniae*; furthermore, production of IL-22 by ILC3s could also help to control the infection.

### IL-17 from ILC3s Could Be Important in the Fight Against *Pseudomonas aeruginosa* Infection

*P. aeruginosa* is a common gram-negative bacterium capable of colonizing a wide range of ecological niches (49). It was previously discovered that exposing mice to *C. albicans* reduced lung injury and bacterial burden from a posterior *P. aeruginosa* infection (50), mainly because *C. albicans* increased the production of IL-22 from ILCs, promoting production of antimicrobial peptides and thus, *P. aeruginosa* protection (51). Moreover, it has recently been demonstrated in a murine *P. aeruginosa pneumonia* model that IL-22 upregulated IFN-λ expression and reduced recruitment of neutrophils, which provided a better outcome in the lung pathology of the mice compared with the exacerbated lung inflammation and pathology in mice with IFN-λ or IL-22 neutralization (52). In addition, further data showed that 90% of IL-17 production in *P. aeruginosa* infection appeared to come from ILC3s rather than CD3^+^ lineages, including γδ T cells (**Figure 3**). IL-17 was shown to be important for this infection since *Il-17ra^−/−^* mice presented greater pulmonary bacterial loads at 2 weeks following infection by YH5 strain, and with the NH57388A strain, all of which died. However, there is a need for further characterization with CD127 and RORγt as ILC3 markers, in order to be sure of the identity of these IL-17^+^ CD3^low-int^ CD19^-^ CD11c^-^ NK1.1^-^ cells (53). To conclude, in a *P. aeruginosa* infection, IL-22 is important for a controlled response and IL-17 is necessary in order to reduce the bacterial load. However, there is still a need to characterize ILC3s in this regard.

### Reduced Numbers of ILC3s Provoke a Greater *Paracoccidioides brasiliensis* Load in *Ahr^−/−^* Mice

The genus paracoccidioides (*P. brasiliensis* and *P. lutzii*) is a dimorphic fungus that causes paracoccidioidomycosis (PCM), which is a systemic granulomatous mycosis (54). Recent studies of *P. brasiliensis* infection in *Ahr* ^−/−^ mice have revealed a reduction of the cytokines, indoleamine 2,3-dioxygenase 1 (IDO-1) expression and kyneurine (Kyn) synthesis by pulmonary DCs. This altered pulmonary microenvironment provided a poorer outcome, a greater fungal load and a decrease in the number of NK and ILC3s, with no differences in the ILC1 or ILC2 populations (55) (**Figure 3**). However, there exists a need to address the potential role of ILC3s in defending against *P. brasiliensis* with the use of more specific transgenic mice.

## The Repercussion of Blood or Systemic Infections in ILC3 Population

### Immunodeficiency Viruses Reduce ILC3s in Infected Subjects

Human immunodeficiency virus (HIV) is a retroviral RNA virus that attacks the immune system; it is characterized by a systemic chronic inflammation and if not treated, it can lead to the development of acquired immunodeficiency syndrome (AIDS) (56). Studies published to date on HIV and Simian Immunodeficiency Virus (SIV) infections suggest that there is an early and sustained reduction of ILC numbers, as well as diminished function thereof, most notably ILC3s in the gut; this causes disruption of the mucosal barrier and immune dysregulation. A better understanding of the mechanisms of loss and dysfunction is relevant with regard to designing new immunotherapies for restoring these cells. Different mucosal NK-cell subpopulations were studied in rectal tissue of macaques chronically infected with SIV, finding a reduction of the NKp44^+^ NK (ILC3s) population and alteration of its functional profile resulting from diminished IL-17 secretion due to IDO1 catabolites, which were secreted by DCs (57). In a subsequent investigation with an HIV model of humanized mice, ILC3s became depleted as a result of CD95 induction in ILC3s by HIV, *via* a plasmacytoid DC- and IFN-I-dependent mechanism that sensitized ILC3s to undergo CD95/FasL-mediated apoptosis. It was therefore hypothesized that the lack of IL-22 was one of the reasons why the intestinal barrier function was impaired in HIV patients (58–60). On the other hand, other studies suggest that the loss of ILC3s in lymphoid tissues during SIV infection might be due to the induction of apoptosis by microbial products through the toll-like receptor 2 (TLR2) (lipoteichoic acid) and TLR4 (LPS) pathways (61). More recently a study of simian-human immunodeficiency viruses (SHIV), which explored breastfeeding-related HIV infection in newborns, found that infection caused depletion of ILC3s in the gut. The infection altered the trafficking and chemokine receptors in ILC3s, reducing the expression of α4β7 integrin in colonic ILC3s and consequently minimizing IL-22 secretion in colon and oral mucosa. The aforementioned study corroborated the premise that the lentivirus infection could have significant effects on the frequency, phenotype, and function of innate cells in the oral and gut mucosa such as the reduction of ILC3 IL-22^+^ which might be particularly relevant in infants infected through breastfeeding, because their immune system is not fully functional (62). Importantly, it has recently been demonstrated that NKp44^+^ ILCs play a protective role in the progression of the disease, as their higher frequency in the mucosa was associated with delayed SIV acquisition and decreased viremia in vaccinated macaques (63), thus confirming previous published data (64).

In human subjects it was found that all ILCs in blood were depleted during infection, but surprisingly a reduction of ILC numbers in the gut was not detected (65). Recently, however, depletion of ILCs has been found in the blood, but this has also been detected in the gut of people with HIV-1, even with effective antiretroviral therapy (ART). It was demonstrated *ex vivo* that inflammatory cytokines associated with HIV-1 infection irreversibly disrupted ILCs and expanded Transcription Factor 7 (TCF7)-dependent memory NK cells, thus explaining the chronic inflammation in people with HIV-1 (66). Interestingly, preterm birth was also associated with lower levels of all three peripheral ILC subsets in pregnant HIV positive women (67). Moreover, vertically HIV-infected children also exhibited a loss of all circulating ILCs which, unlike CD4^+^ T cells, were not restored by long-term ART if not initiated at birth (68). Further research is required in order to study the specific consequences of the lack of ILC3s in HIV-infected patients. In summary, ILC3s are depleted in the blood and gut of HIV and SIV infected human and macaque subjects potentially *via* the CD95 induction or TLRs pathway.

## Defensive Role of ILC3s in Gut

### Several Transcription Factors Expressed in ILC3s Are Essential in the Fight Against *Salmonella* and the Possible Subsequent Development of Intestinal Fibrosis

**Figure 4:**
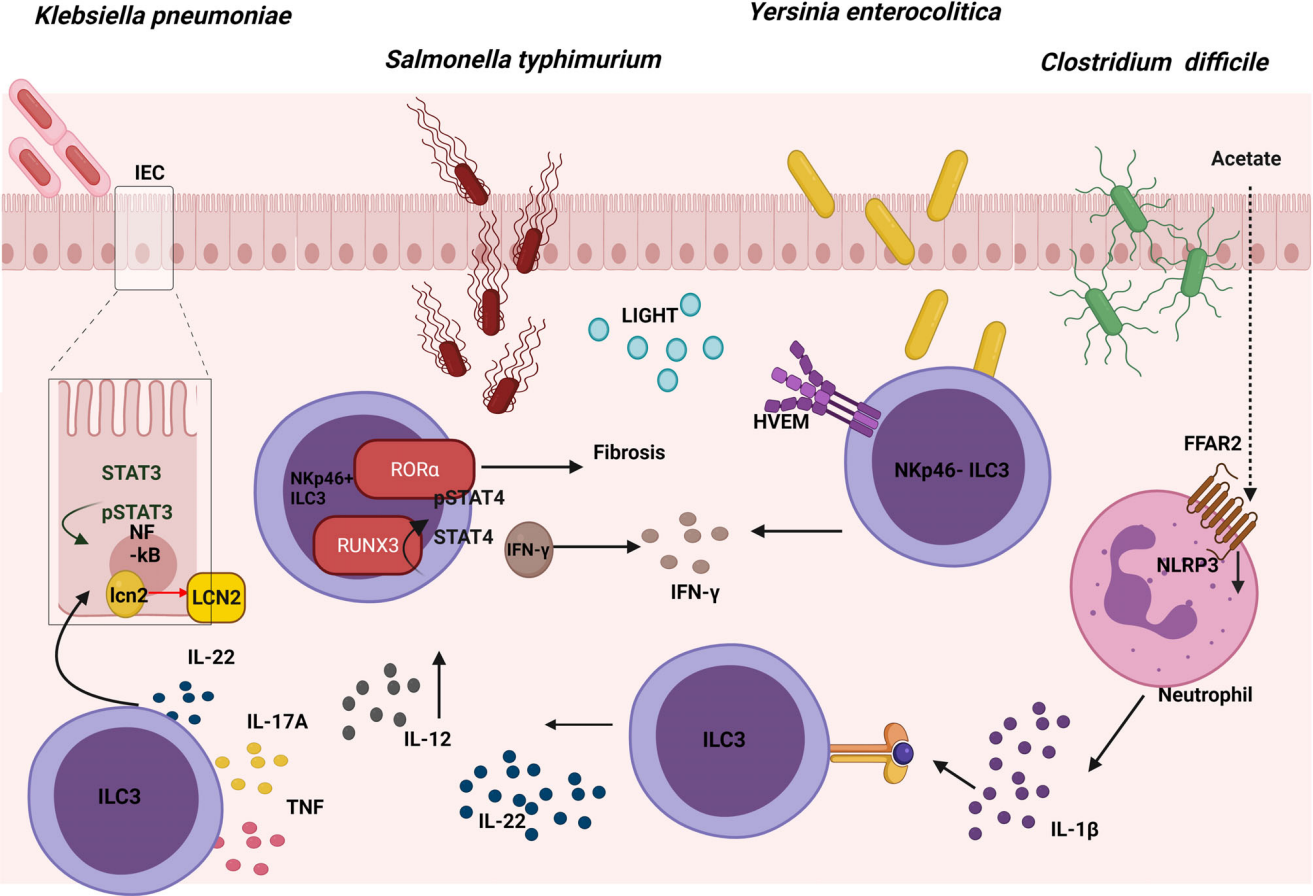
Role of ILC3s in the response against gut pathogens. The expression of RORα in ILC3s leads to intestinal fibrosis in a Crohn’s disease model of *S. Typhimurium*. At the same time, an up-regulation of RUNX3 expression in ILC3s promotes the IL-12/STAT4/IFN-γ signaling pathway, which is also involved in the fight against this pathogen. Cytokines produced by ILC3s promote the NFkB and STAT3 pathway, both of which are involved in the production of lipocalin-2 (LCN-2) by intestinal epithelial cells (IECs), inhibiting the growth of *K. pneumoniae*. The binding between LIGHT and its receptor HVEM in ILC3s promotes IFN-γ production which is involved in the defence against *Y. enterocolitica*. In the defence against *C. difficile*, acetate promotes the production of IL-1β in neutrophils *via* the FFAR2-NLRP3 inflammasome axis, activating ILC3s *via* IL-1R to produce IL-22.

*Salmonella enterica* serovar Typhimurium (*S. typhimurium*) is an enteropathogenic bacterium that causes inflammation in both the small and large intestine (69). Salmonellosis comprises major food-borne gastrointestinal diseases caused by the Salmonella species, and most infectious serovars cause enterocolitis or diarrhea. Mice lacking hematopoietic expression of RORα were protected from intestinal fibrosis in a Crohn’s disease model of *S. typhimurium* because ILC3s produced less IL-17A. Reconstituting lethally irradiated mice with equal numbers of bone marrow (BM) cells from *Rag1^−/−^* mice combined with either WT or *Rora* ^sg/sg^ BM cells, showed that RORα-expressing ILC3s were sufficient to cause fibrosis (70) (**Figure 4**). In addition, diminished expression of a number of ILC3-defining transcripts in the cecum and mLN was observed in *Salmonella*-infected *Rora* ^sg/sg^ BM transplant chimeric mice using single-cell RNA sequence analysis compared with their WT counterparts. These cells, known as ex-RORγt ILC3s, were similar to ILC1s and promoted *Salmonella* and IFN-γ–mediated enterocolitis. Therefore, even after chronic *Salmonella* infection, RORα is still essential with regard to conserving the role of ILC3s in peripheral tissues and their lineage fate (71). In summary, these results show that expression of RORα promotes susceptibility to ﻿intestinal fibrosis due to an increase in ILC3 responses.

Following oral infection with *S. typhimurium* a significant increase was observed in the number and percentage of Nkp46^+^ILC3 cells in the lamina propria (LP) of the murine small intestine, with elevated levels of Runx3 (Runt-related transcription factor 3). To study the role of this transcription factor during infection in these cells, *Runx3* ^fl/fl^ *Plzf* Cre (*Runx3* cKO) mice were generated; these proved to be much more susceptible to *S. typhimurium* intracellular bacterial infection. Promyelocytic leukemia zinc finger protein (PLZf) is expressed by ILCs during their development, as well as in Th1, CD8^+^ T cells and several invariant T cells. Flow cytometry was employed to demonstrate that part of the inability to control the infection was because IFN-γ secretion decreased in Nkp46^+^ILC3s. Previously, it was reported that after binding IL-12 to IL-12R, STAT4 proteins were phosphorylated and accumulated in the promoter areas of IFN-γ to induce its expression. Thus, to analyze the role of this axis, sorted Nkp46^+^ILC3s from *Runx3* cKO mice were stimulated with IL-12; by means of flow cytometry a decrease was seen in pSTAT4 compared with WT, which demonstrated that these cells had impaired the IL-12/STAT4 signaling. Moreover, in order to understand the underlying mechanism, chromatin immunoprecipitation (CHIP) analysis was used to show that RUNX3 was directly bound to the promoter region and intron 8 of the IL12Rβ2 gene (72). In conclusion, upon infection with *S. typhimurium*, Nkp46^+^ILC3s upregulated Runx3 expression, which promotes the IL-12/STAT4/IFN-γ signaling pathway, and which would in turn limit intracellular bacterial infection (**Figure 4**).

### *Giardia Lamblia* Infection Produces IL-17-Expressing ILC3s in Gut

*Giardia lamblia* is an extracellular protozoan pathogen that inhabits the human small intestine, causing diarrhea (73), which is mainly due to food contamination; it can cause foodborne diseases including giardiasis (74). It has been found that *G. lamblia* increased the secretion of IL-17A, IL-17F, IL-1β of isolated ILCs from the LP of mouse small intestine *in vitro*. Moreover, mice inoculated with *G. lamblia* trophozoites showed more IL-17-expressing ILC3s in the LP of mouse small intestine. Therefore, IL-17-producing ILC3s could be key players upon infection with *G. lamblia* (75) but further research studying this model in *Rag^−/−^* mice with the depletion of *Il-17* gene in the ROR*γ*t^+^ cells is required to demonstrate this hypothesis.

### ILC3-Derived Cytokines Induce Lipocalin-2 Expression in Epithelial Cells Which Inhibits the Growth of *Klebsiella pneumoniae*

*K. pneumoniae* is an opportunistic pathogen commonly related to lung infections, and studies in recent years are beginning to associate it with gastrointestinal tract-related diseases (76). As a response to this infection in the gut, a release occurs of epithelial-derived antimicrobial factors produced by intestinal epithelial cells (IECs). In particular, lipocalin-2 (LCN-2) provides a specific protection against the Enterobacteriaceae family. Furthermore, one of the cell types playing an important role in the regulation of antimicrobial responses in IECs involves ILC3s; in particular, these act by secreting IL-22 during enterobacteriaceae infections. It was demonstrated that IL-22 regulated the expression and production of LCN-2 in IECs which was capable of inhibiting the growth of *K. pneumoniae in vitro*. The supernatant of NKp44^+^ and NKp44^-^ ILC3s culture isolated from human tonsil samples and stimulated with IL-2, IL-1β, and IL-23, was able to increase expression of LCN-2 in an epithelial cell line *in vitro*. The importance of IL-22 has been confirmed by the use of anti-IL-22 Mab, which reverted LCN-2 expression and protein release in IECs *in vitro*. All these data supported the premise that ILC3-derived IL-22 could enhance the expression of LCN-2 in human IECs (77) (**Figure 4**). The role of other cells in this mechanism remains to be established, and the next step should involve demonstrating this mechanism in an *in vivo* model. To this end, neonatal mice exposed to a short (SE) or long (LE) exposure of broad-spectrum antibiotics were examined, thus observing that the LE mice were more susceptible to *K. pneumoniae*-induced sepsis at 2 weeks of life. Appreciably, the percentage of ILC3s and their capacity to produce IL-17A in the LP showed a decrease in the LE mice and were capable of partially rescuing the physiological phenotype by means of reconstitution with mature microbiota (78). This study supported a role for microbiota dependent suppression of IL-17A-producing ILC3s in increasing susceptibility to late-onset sepsis. In conclusion, cytokines produced by ILC3s can regulate the intestinal epithelial cells in order to defend against *K. pneumoniae.*

### ILC3-Derived IFN-γ Production Protects Against *Yersinia enterocolitica* Infection

*Y. enterocolitica* is a gram-negative bacterium that produces enterocolitis and can cause infection by ingestion of contaminated food, particularly raw or undercooked pork products, and of unpasteurized milk (79). Remarkably, *Ahr* ^fl/fl^ *Rorc* Cre mice were used to demonstrate that ILC3s play a significant role in the defence against *Y. enterocolitica*. In order to investigate which ILC3 subset was necessary, *Rorc* ^fl/fl^ *Nkp46* Cre mice were infected. This showed that the NKp46^+^ ILC3 subset was not required to mediate early host defence. Interestingly, the survival, weight loss and areas of necrosis were rescued by performing an adoptive transfer of NKp46^-^ ILC3 into *Rag2^−/−^cγc^−/−^* infected with *Y. enterocolitica*. However, the same result was obtained transferring equal numbers of NKp46^+^ ILC3s, indicating that these cells also had a protective capacity in immunosuppressive mice but were redundant in immuno-competent mice [also demonstrated in *C. rodentium* infection model (80)]. By contrast, recipient mice injected with CCR6^+^ ILC3s sorted from LP of the small intestine (LTi-like cells) were not protected. In addition, bacterial translocation in *Ifng* ^−/−^, IL-17Rra^−/−^, and IL-22^−/−^ mice after oral *Y. enterocolitica* infection showed that IFN-γ was required for host defence, especially those produced by NKp46^-^ ILC3. An adoptive transfer of CCR6^−^ ILC3s from *Ifng^−/−^* or WT mice in *Rag2^−/−^ cγc^−/−^* mice confirmed the importance of IFN-γ production by ILC3s, but, surprisingly, IL-17 and IL-22 cytokines had little effect on the outcome. Previous work suggested that the herpesvirus entry mediator (HVEM) expression was important as a regulator of the mucosal immune system in multiple cell types (81); it was expressed by all small intestine ILC subsets in mice and all ILCs in human peripheral blood. Therefore, *Hvem* ^−/−^, *Hvem* ^fl/fl^ *Rorc* Cre, and *Hvem* ^fl/fl^ *Cd4* Cre mice were infected, showing that ILC3s (but not CD4^+^T cells expressing HVEM) were essential for the early protection of *Y. enterocolitica*. Indeed, RORγt-mediated deletion of HVEM only decreased IFN-γ secretion from ILC3s. The essential role of HVEM in ILC3s was confirmed by the poorer outcome of an adoptive transfer of *Hvem* ^−/−^ ILC3s into *Rag2^−/−^ cγc^−/−^* mice infected with *Y. enterocolitica*, in comparison with the adoptive transfer of WT ILC3s. IFN-γ production by ILC3s increased through a HVEM ligand known as LIGHT, at both the transcript and protein levels. All these data showed that HVEM signaling mediated by LIGHT plays a critical role in regulating ILC3-derived IFN-γ production for protection against *Y. enterocolitica* following infection (82) (**Figure 4**). In summary, the signature ILC3 cytokines, IL-22 and IL-17A, are not important for protection against *Y. enterocolitica*, but the IFN-γ produced by these cells does play an important role.

### Acetate-Induced IL-22 Secretion by ILC3s Could Help to Defend Against *Clostridium difficile*

*C. difficile* is an opportunistic anaerobic gram-positive bacterium that causes most nosocomial antibiotic–associated diarrheas and colitis. During colonization, pathogenic strains of *C. difficile* produce 2 exotoxins that induce acute inflammation, cell necrosis, and fluid secretion in/into the gastrointestinal tract (83). The role of ILC3s in the defence against *C. difficile* was studied in the murine model and although upregulation of the expression of ILC1- or ILC3-associated proteins in the large intestine was detected following *C. difficile* infection; selective loss of ILC3s (*Rorc* ^−/−^ mice) or ILC3 effector molecules (*Il-22^−/−^* and *Il-17* ^−/−^ mice) did not affect the survival or the severity of the infection. This would appear to suggest that ILC3s made a minor contribution to resistance, while the loss of IFN-γ or T-bet-expressing ILC1s in *Rag1* ^−/−^ mice increased susceptibility to *C. difficile* (84). However, according to the research conducted by J. L. Fachi, who investigated whether hypoxia could regulate the activation of ILC3s, hypoxia inducible factor-1α (HIF-1α)-deficient RORγt mice infected with *C. difficile* presented more severe infection compared with their WT counterparts, although they displayed significantly lower numbers of gut ILC3 and higher numbers of ILC1s (85). The aforementioned group previously investigated previously the mechanism through which oral administration of acetate enhanced the host’s resistance to *C. difficile*. Acetate-treated mice showed a moderate increase in the number of ILC3s (but not that of other ILCs) in the gut and increased expression of RORγt and IL-22 specifically in colon. In addition, free fatty acid receptor (*Ffar*)2^−/−^ and *Rag2^−/−^ cγc^−/−^* transgenic mice presented a more severe infection, although acetate was not triggering the expression of IL-22 through FFAR2 directly in ILC3s *in vitro*. As neutrophils also expressed FFAR2, acetate actually stimulated production of IL-1β through the FFAR2-NLRP3 inflammasome axis in these cells, thus activating ILC3s through IL-1R (86) (**Figure 4**). Therefore, acetate supplementation improves protection against *C. difficile* in colon by inducing the production of IL-22 in ILC3s *via* FFAR2/IL1-β axis in neutrophils. Considering the controversy of the possible role of ILC3s in the defense against *C. difficile*, there is a need for further study with other transgenic mice in order to elucidate the role play by ILC3s in this infection model.

### An Increase in IL-22-Producing ILC3 Cells Following Exposure of *Dectin-3^−/−^* Mice to *Candida albicans* Promotes Colitis-Associated Colon Cancer

**Figure 5:**
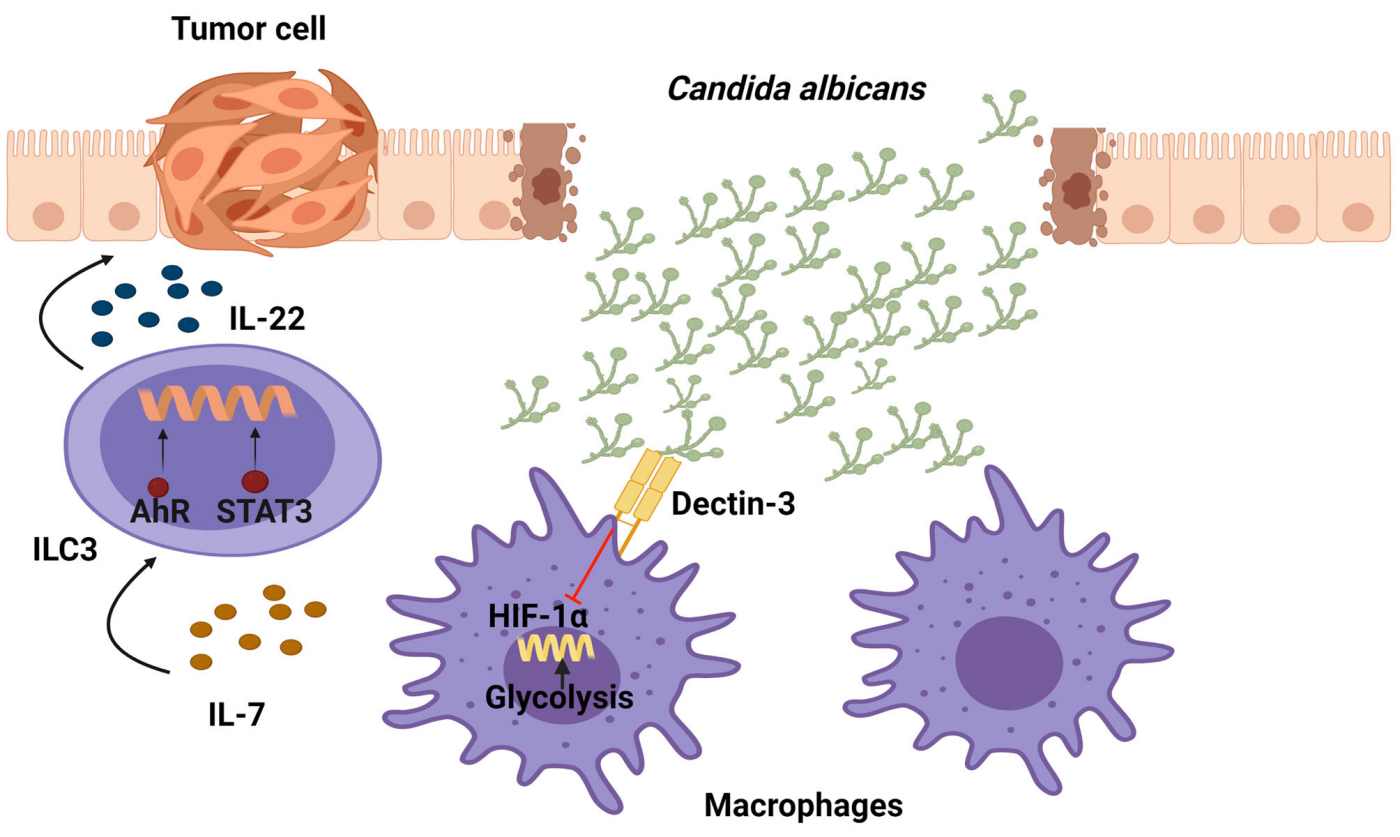
Function of IL-22-producing ILC3 cells in the development of colitis-associated colon cancer (CAC) following exposure of *Candida albicans*. Dectin-3, expressed by myeloid cells, recognizes this fungus and down-regulates the production of IL-7. In Dectin-3-deficient mice, macrophages display a HIF-1α-dependent glycolysis which brings ILC3s to produce IL-22 (through STAT3 and AhR), leading to the progression of CAC.

Among the fungi that resides in the gastrointestinal tract of healthy individuals, Candida is the dominant one. In order to sense a fungi infection and induce an activation of downstream signaling, on their surface pattern innate immune cells have recognition receptors such as mammalian C-type lectin receptors. In particular, Dectin-3 receptors recognize α-mannans on the surface of multiple fungi such as *C.albicans*, *Paracoccidioides brasiliensis*, and *Cryptococcus.* Interestingly, *Dectin-3* ^−/−^ mice have recently been found to present enhanced colitis-associated colon cancer (CAC) correlating with an increase in the *C. albicans* burden. The *Dectin-3* ^−/−^ mice recruited more macrophages and ILC3s to mesenteric LNs and LP tissues than the WT tumor-bearing mice. Regarding the mechanism, it was shown that an elevated *C. albicans* load triggered glycolysis and IL-7 production through a HIF-1α-dependent pathway in macrophages from LP in *Dectin-3* ^−/−^ mice. Importantly, IL-7 induced IL-22 secretion in sorted ILC3s *in vitro via* AhR and STAT3. In conclusion, an increase in IL-22 production by ILC3s through STAT3 and AhR promotes CAC progression as a result of a Dectin-3 deficiency in macrophages, which increases IL-7 production through HIF-1α-dependent glycolysis (87) (**Figure 5**).

### Several Signals Can Regulate ILC3 Responses Against *Citrobacter rodentium*

*C. rodentium* is a gram-negative mucosal pathogen that shares several pathogenic mechanisms with enteropathogenic *Escherichia coli* (EPEC) and enterohemorrhagic *E. coli* (EHEC); it therefore constitutes a useful murine model for investigating infectious colitis as well as intestinal bowel disease (IBD), enabling an assessment of the mucosal immune responses, host-pathogen interactions and wound healing, among others (88). Previous field studies have demonstrated that ILC3s plays a key role in the control of *C. rodentium* infection by secreting large amounts of IL-22 and IL-17 (11, 13, 89). However, some studies support the idea that Nkp46^+^ ILC3s prove to be redundant for control of *C. rodentium* infection in the presence of T cells; these studies involved the use of transgenic mice with conditional deletion of the key ILC3 genes *Stat3*, *Il-22*, *Tbx21* and *Mcl1* in Nkp46-expressing cells (80). In recent years, different signals have been found to regulate ILC3 responses against *C. rodentium*.

Hypoxia: ILC3s have been observed to undergo metabolic reprogramming combining glycolysis with mitochondrial ROS production to fight *C. rodentium* infection. *Rag1^−/−^* mice lacking the gene for the mTOR complex 1 (mTORC1) subunit Raptor in RORγt-expressing cells, or WT mice treated with rapamycin, showed greater susceptibility to *C. rodentium* infection. Significantly, *Raptor* ^fl/fl^ *Rorc* Cre mice presented much lower numbers of ILC3s at steady state suggesting a possible role in haematopoiesis although this possibility was not addressed in the aforementioned research. To study whether mTORC1-HIF-1α could drive an impact in ILC3 function, ILC3 cell line MNK3 and primary intestinal ILC3s were activated *in vitro* and incubated with mTORC1 and HIF-1α inhibitors, showing a decrease in IL-17A and IL-22 production, as well as proliferation. Therefore, the mTORC1 pathway could sustain ILC3s activation, cytokine production, and proliferation, but there is a need to conduct further studies in order establish the function of mTORC1-HIF-1α pathway in *C. rodentium* infection *in vivo* (90).

**Figure 6:**
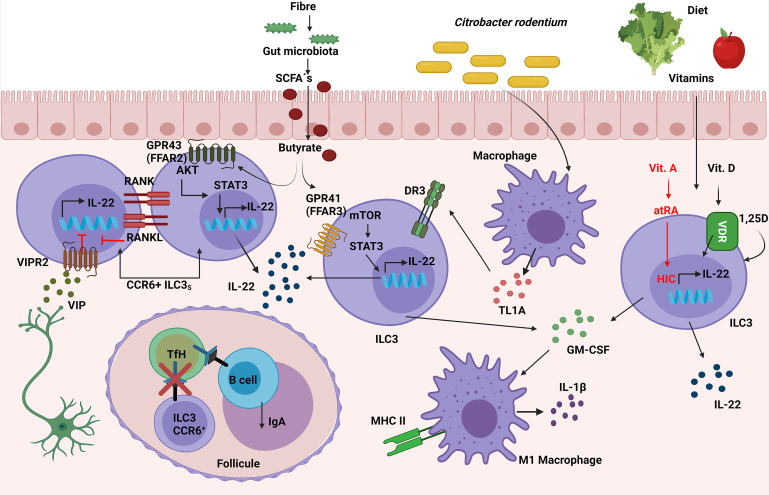
Role of ILC3s in the response against *C. rodentium*. Vasoactive intestinal peptide (VIP), released by neurons in lymphoid patches in close contact with CCR6^+^ ILC3s, inhibits their IL-22 secretion. Simultaneously, ILC3s have a negative intrinsic modulation, expressing both RANK and RANKL, which suppresses the induction of IL-22 and IL-17. Susceptibility to *C. rodentium* infection is caused by these inhibitory mechanisms. The interaction between CCR6^+^ ILC3 and TfH cells through MHC-II in the mesenteric lymph nodes limits the TfH responses as well as pathogen specific-IgA. Simultaneously, ILC3s expression of TL1A receptor DR3 confers protection against *C. rodentium*. SCFAs produced by the commensal microbiota from dietary fibers increase IL-22 secretion in ILC3s *via* the FFAR2/FFAR3-AKT/mTOR/STAT3 molecular pathway, thus contributing to the elimination of the infection. Moreover, ILC3-derived GM-CSF production can improve antimicrobial responses by increasing the expression of IL-1β and MHC-II in macrophages (M1-like macrophages). Furthermore, Vitamins D and A in the diet increase ILC3’s synthesis of IL-22, which protects against *C. rodentium*; vitamin A’s molecular mechanism is *via* HIC (Hypermethylated in cancer 1).

Microbiota: Microbiota-derived short-chain fatty acids (SCFAs) promoted IL-22 production by CD4^+^ T cells and ILC3s both *in vitro* and *in vivo*, resulting in a significant inhibition of *C. rodentium*-colitis. In addition, *in vitro* assays with G protein-coupled receptor (GPR)41(FFAR3)-specific agonist and other inhibitors showed that butyrate promoted IL-22 production through the FFAR3, activating HIF-1α, AhR, STAT3 and mTOR proteins. In contrast, IL-22 was induced by butyrate at similar levels in WT and GPR109a-deficient or GPR43(FFAR2)-deficient CD4^+^ T cells, suggesting that it was particularly dependent on GPR41 (91). However, the deficiency of FFAR2 in RORγt-expressing cells caused a decrease in *in situ* proliferation and IL-22 production, specifically in colonic CCR6^+^ILC3s (and not in Nkp46^+^ ILC3s) which led to increased susceptibility to *C. rodentium* infection in *Ffar2* ^fl/fl^ *Rorc* Cre mice. Additionally, FFAR2 agonists increased ILC3-derived IL-22 *via* an AKT and STAT3 axis *in vitro* (92). Interestingly, neither alterations in cell frequency nor IL-22 nor IL-17A production in colonic RORγt^+^ CD4^+^ T cells from *Ffar2* ^fl/fl^ *Rorc* Cre mice were observed, as suggested the data published by Yang in CD4^+^ T cells (91). In this regard, it has recently been published that SCFAs, produced by the commensal microbiota from dietary fibres (DF), supported optimal expansion of all ILCs *via* both FFAR2 and FFAR3 receptors. Mice fed with a DF diet increased the number of ILC3s in the gut but not in the lymphoid organs, improving intestinal immunity to *C. rodentium* infection. In addition, the signaling triggered by SCFAs on ILCs was analyzed *in vitro*, showing an activation of Phosphoinositide 3-kinase (PI3K), STAT3, STAT5, and mTOR, which is important for ILC proliferation. Furthermore, FFAR2 was required for normal expansion of ILC1s and ILC3s both in the intestine and in systemic tissues following *C. rodentium* infection; this was demonstrated with the use of *Ffar2^−/−^* mice (93) (**Figure 6**). To summarize, the protection against *C. rodentium* provided by IL-22 secreting-ILC3 cells can be activated by FFAR2 and FFAR3 triggered by microbiota-derived SCFAs.

Diet: Vitamins can also regulate innate responses to infections. Vitamin A metabolite all-trans-retinoic acid (atRA) has been implicated in the regulation of immune tolerance to food antigens, partially by regulating ILC responses in the intestine. The role of the transcription factor Hypermethylated in cancer 1 (HIC1), previously identified as an all-trans retinoic acid (atRA) responsive gene in intestinal Th cells, was essential for the regulation of ILC3s in the defence against *C. rodentium*, because *Hic*1^flfl^ *Rorc* Cre mice treated with depleted anti-CD4 Mab were more susceptible to the infection and showed less RORγt^+^ T-bet^+^ ILC3s in LP (94). The role of vitamin D in the clearance of *C. rodentium* infection was tested in Cyp27B1 (vitamin D 1α-hydroxylase mimicking Vitamin D deficiency) KO mice previously fed with a low vitamin D diet (D-), making them more susceptible due to lower levels of IL-22 from both ILC3s and Th17 than mice with a vitamin D supplemented diet (D+). ILC3s were important with regard to maintaining the defence against *C. rodentium*, because *Rag^−/−^* mice on a D- diet presented lower levels of IL-22 from ILC3, and therefore had more severe colitis and a higher mortality than their D+ diet littermates. Furthermore, this phenotype was rescued by administering 1,25D which raise ILC3s and IL-22 levels, or by administering IL-22Fc fusion protein IL-22 *in vivo*, thus proving that vitamin D was required for healthy regulation of ILC3 response against *C. rodentium* (95) (**Figure 6**). In short, vitamins A and D are are needed to maintain suitable levels of IL-22 secretion from ILC3s in order to enhance the protection against *C. rodentium*.

Nervous system: Previous studies have associated ILC3s with the mucosal neural system (16). It was demonstrated that ILC3s expressed circadian clock genes and IL-17/IL-22-producing ILC3s diminished significantly in *Arntl* ^fl/fl^ *Vav1* Cre and the *Arntl* ^fl/fl^ *Rorc* Cre mice (conditional deletion of the master circadian activator aryl hydrocarbon receptor nuclear translocator-like gene *Arntl* in hematopoietic cells and RORγt-expressing cells respectively), showing greater susceptibility to *C. rodentium*. This ILC3 reduction in the gut was not due to an alteration of the numbers of common lymphoid progenitors but rather involved an impairment of the migration of ILC3s to LP, because ILC3s from *Arntl* ^fl/fl^ *Rorc* Cre mice displayed reduced expression of CCR9, an essential chemokine receptor for intestinal LP migration. In general terms, circadian clock genes play a significant role in the migration and regulation of ILC3s for fighting against *C. rodentium* (96).

As for the modulation of ILC3s by the nervous system, CCR6^+^ ILC3 which expressed neural-related genes such as the vasoactive intestinal peptide receptor 2 (VIPR2) were in close proximity with VIP^+^ neurons in the cryptopatches and lymphoid patches of ileum and colon. VIPR2^−/−^ CCR6^+^ ILC3s generated more IL-22, which was inhibited by using VIPR2 agonists, suggesting that VIP inhibited the production of IL-22 in ILC3s. Additionally, with the use of DREADD mice (designer receptors exclusively activated by designer drugs in VIP^+^ cells), VIP neurons were shown to modulate CCR6^+^ ILC3 cytokine secretion through VIPR2, which is key in the protection against *C. rodentium*. The DREADD inhibition of VIP^+^ neurons provided protection against *C. rodentium*; this is in contrast with DREADD activation in the presence of *C. rodentium*, which led to severe disease that could be reversed with the administration of recombinant mouse IL-22, indicating that activation of VIPergic neurons may be contributing to intestinal barrier malfunction (97) (**Figure 6**). Thus, the neuron system regulates ILC3 response to *C. rodentium* by the modulation of Vipergic neurons.

Cytokines or chemoattractant: The primary source of tumor necrosis factor-like cytokine 1A (TL1A) in the gut are CX3CR1^+^ MNPs and interestingly, *Rag2* ^−/−^ *Tnfsf25* ^fl/fl^ *Rorc* Cre (RORγt^+^ cells deficient for TL1A receptor DR3) mice infected with *C. rodentium* lost more weight and presented lower survival rates compared to their littermate controls, thus revealing a protective mechanism of TL1A in ILC3s during acute colitis (98) (**Figure 6**). As for chemotactic receptors, it has been shown that GPR183, which is normally expressed by follicular B cells, DCs and CD4^+^ T cells, was also highly expressed in ILC3s. Additionally, GPR183 was found to be an important receptor for the migration of ILC3s towards its ligand 7a,25-OHC (produced by stromal cells); this was revealed by means of a transwell migration assay *in vitro* with sorted GPR183^−/−^ ILC3s. Moreover, *Gpr183LacZ*/+ (KO reporter mice) mice exhibited lower numbers of ILC3s and fewer cryptopatches, a fact which made them more susceptible to colitis by *C. rodentium* infection, demonstrating that GPR183 and 7a,25-OHC controlled the distribution and accumulation of ILC3s in the mLNs and Peyer’s patches (99). Therefore, GPR183 plays an essential role in terms of migration and distribution of ILC3 and consequently in a *C. rodentium* infection. As for TL1A, it would be of general interest to study in greater depth the molecular pathway involved in the protection against *C. rodentium*.

Membrane proteins: NKR-P1A, expressed in human NKs, is a type II transmembrane protein belonging to the C-type lectin superfamily and which can recognize C-type lectin-related (Clr) proteins. E. Abou-Samra et al. found that ILC3s in the gut also expressed NKR-P1B (homologue of NKR-1PA in mice). Although NKR-P1B-deficient mice showed higher numbers of ILCs and *γ*δT cells in steady state, lower levels of IL-22 were observed. Moreover, upon *C. rodentium* infection NKR-P1B-deficient mice had more CFUs showing lower levels of IL-22 production from CCR6^+^ ILC3s than their control WT mice. Similar results were obtained in *Rag1* ^−/−^Nkr-p1b^−/−^ mice demonstrating the importance of NKR-P1B in ILC3s in the context of infection (100). As a result, the NKR-P1B ILC3 membrane protein is required to regulate ILC3 numbers and function, although further research on the molecular mechanism is needed.

Another molecular regulation of ILC3s is based on the receptor activator of nuclear factor κ B (RANK) interaction with RANK Ligand (RANKL). A novel negative intrinsic regulation of production of IL-22 and IL-17 by ILC3s was discovered by generating *Tnfrsf11a* ^fl/fl^ *Rorc* Cre (RANK ligand deficient mice in RORγt-expressing cells) and *Tnfrsf11* ^fl/fl^ *Rorc* Cre (RANK deficient mice in RORγt^+^ cells). *Tnfrsf11* ^fl/fl^ *Rorc* Cre mice exhibited hyperresponsive CCR6^+^ ILC3s and increased IL-17/IL-22 production, presenting less severe colitis upon *C. rodentium* infection. *Rag1* ^−/−^ *Tnfrsf11* ^fl/fl^ *Rorc* Cre mice had a similar outcome of the disease demonstrating that the regulation of RANK-RANKL was in ILC3s and not in T cells (101) (**Figure 6**). Several years ago, AhR was shown to constitute a relevant transcription factor relevant for combating *C. rodentium*, as knocking out AhR resulted in a loss of IL-22^+^ ILC3s (4, 102, 103). It has recently been determined that AhR expression in ROR**γ**t^+^ cells alone was sufficient with regard to maintaining a functional ILC3 compartment and controlling *C. rodentium* infection; this was demonstrated by the complete protection of *Ahr* ^CAIR/CAIR^ *Rorc* Cre mice, an AhR-knockin mouse model that expressed a constitutively active form of AhR (CA-AhR) only in ROR**γ**t^+^ cells (104). Conclusively, AhR is required for ILC3 to produce IL-22 in the presence of *C. rodentium*, and ILC3s possess intrinsic self-regulation of their immune response, which is mediated *via* the RANK-RANKL interaction

ILC3s can regulate other immune cells during *C. rodentium* infection through GM-CSF secretion. Mice lacking ILCs and T cells (*Rag1/2* ^−/−^ treated with CD90.2 Mab) were more susceptible to *C. rodentium* infection; they presented an accumulation of immature monocyte in gut following dextran sulfate sodium colitis and a reduction of pro-IL-1β expression in intestinal mononuclear phagocytes. As ILC3s were the major GM-CSF-producing cell type in the colon during colitis, sorted RORγt^+^ ILC3s or ILC3-conditioned media were co-cultured with BMDM, resulting in an enhancement of the IL-1β, MHC-II expression in BMDM and suppression of IL-10 (characteristics of M1 macrophage). Importantly, these effects were inhibited by the addition of a GM-CSF-neutralizing antibody. In addition, an increase was also observed in the expression of genes associated with M2 macrophage activation in colonic macrophages of ILC-depleted mice *in vivo*. Consequently, these data reveal that GM-CSF-mediated ILC3-macrophage crosstalk play a vital role in calibrating the intestinal macrophage phenotype to enhance anti-bacterial responses (105) (**Figure 6**).

It has been shown that Lti-like ILC3s might also be interacting with follicular T helper cells (TfH) in the interfollicular region of mesenteric lymph nodes, thus limiting the TfH response and B cell-derived IgA production against mucosal microbiota as well as B cell class switching through MHC-II antigen presentation in steady state. This interaction between Lti-like ILC3s and TfH cells was also relevant in *C. rodentium* infection, which was demonstrated with the use of mice lacking MHC-II in ILC3s, observing greater numbers of TfH cells and pathogen-specific IgA after infection with C. *rodentium.* Further studies are required to determine the function of Lti-like ILC3 interactions with TfH and the consequences on TfH function in infection (106) (**Figure 6**).

## Discussion

The importance of cytokine release for combatting pathogens has long been extensively described. The present review addresses the latest studies on ILC3s in the early release of cytokines in various organs of the body in mice and humans - in particular the secretion of IL-17 and IL-22, and their role in the early response to a wide range of pathogens. Although ILC3s have previously been described to be important for defending against extracellular bacteria, recent evidence suggests they can also provide defence against parasites or even viruses. And apart from playing an essential role in the gut, they also have an important function in other organs. Furthermore, the present review highlights the new molecular pathways that have recently been described for the different roles ILC3s play in each organ (**Table 1**); this renders our review a useful tool for further investigation in the field. It is important to highlight that the methodology employed to study the molecular mechanisms involved in the function of ILC3s during infections *in vivo*, entails generating conditional transgenic mice, usually in RORγt-expressing cells, and in order to avoid the contribution of T cells, these mice are crossed with *Rag^−/−^* mice. Consequently, the function of RORγt-expressing cells, which include not only Nkp46^+^ ILC3s or Nkp46^-^ but also Lti cells, has been studied in immunosuppressive mice. Other experimental approaches involve depleting genes in Nkp46^+^ cells; this has led to some groups discovering that Nkp46^+^ ILC3s are redundant during infection in the presence of T cells (immunocompetent mice). In general terms, there is a need for further study of ILC3s and their molecular mechanisms at play in the different diseases of each organ in order to develop new therapies or to make earlier diagnoses.

**Table 1:** Main results of the function and molecular mechanism described in each research article divided by tissue and pathogen.

Tissue	Pathogen	Mechanism	Function	Reference
**SKIN**	*Staphylococcus aureus*	MyD88-dependent induction of IL-1α and IL-36α by KCs. Secretion of IL-17 by both γδ T cells and ILC3s	Skin inflammation and neutrophil infiltration	Nakagawa et al. (20)
**ORAL MUCOSA**	*Candida albicans*	Induction of IL-17 by nTh17s, γδ T cells, and ILC3s	Protection	Gladiator et al. (14), Conti et al. (23), Sparber et al. (22)
**LIVER**	Adenovirus and LCMV	Early IL-17A production by ILC3s	Protection	Jie et al. (25)
	Hepatitis B virus (HBV)	Secretion of IL-17A and IL-22 by ILC3s. Activation of HSC (regulating TGF-β receptor expression and STAT3 pathway)	Fibrosis	Wang et al. (27)
**LUNG**	Influenza virus	IFN-β production. Inhibition of IL-17 production by T cells and potentially by ILC3s	Prevention of secondary bacterial infections	Li et al. (30), Kudva et al. (31), Robinson et al. (32)
		Induction of IL-22+ ILC3s in the lung	Induction of epithelial regeneration and protection against secondary bacterial infections	Ivanov et al. (33), Abood et al. (34)
	*Aspergillus fumigatus*	Induction of IL-22-expressing cells	Inflammation: negative impact on lung function	Reeder et al. (38)
	*Mycobacterium tuberculosis*	CXCR5-dependent iBALT formation through IL-17 and IL-22 secreted by ILC3s	Organization of protective immune responses	Ardain et al. (40)
	*Streptococcus pneumoniae*	Induction of IL-22 by ILC3s	Protection	Van Maele et al. (42)
		Expansion and maturation of pulmonary ILC precursors mediated by IGF-1 from alveolar fibroblasts	Protection	Gray et al. (43), Oherle et al. (44)
		IL-17A expression after IL-7 prophylactic stimulation	Protection	Hassane et al. (45)
	*Klebsiella pneumoniae*	Recruitment of monocytes-producing TNF-α. Induces IL-17-secreting ILCs, which increase ROS in the monocytes	Protection	Xiong et al. (47)
		Secretion of IL-22 by IL-17+ IL-22+ ICOS+ ILC3s	Protection	Iwanaga et al. (48)
	*Pseudomonas aeruginosa*	Production of IL-22 by ILCs induced by previous exposure to *C. albicans*	Induction of antimicrobial peptides and protection against *P.aeruginosa*	Mear et al. (51)
		IFN-λ induction by IL-22	Protection	Broquet et al. (52)
		Induction of IL-17 by innate CD3-CD19- CD11c- NK1.1- cells	Protection	Bayes et al. (53)
	*Paracoccidioides*	IDO-1 expression and Kyn synthesis in DCs induced by AhR	Protection	Araújo et al. (55)
**BLOOD OR SYSTEMIC INFECTIONS**	Simian immunodeficiency virus (SIV)	Reduction of IL-17 secretion due to NKp44+ NK (ILC3s) depletion	Not conclusive	Reeves RK et al. (57)
		Up-regulation of CD95 leads to ILC3s depletion and thus low levels of IL-22	Intestinal barrier impairment	Guo and Fu, (58); Shah et al. (60); Zhang et al. (59)
		Induction of ILC3 apoptosis through TLRs	Not conclusive	Xu et al. (61)
		Reduction of the expression of α4β7 integrin in colonic ILC3s	Not conclusive	Hueber et al. (62)
		Correlation between NKp44+ ILCs levels and delayed SIV acquisition with decreased viremia in vaccinated macaques.	Protection	Rahman et al. (63)
	Human immunodeficiency virus (HIV)	Reduction of ILCs in ART treated people	Not conclusive	Wang et al. (66)
		Correlation between lower levels of ILCs and preterm birth	Not conclusive	Akoto et al. (67)
		Recovery of ILCs in vertically HIV-infected children only when ART is iniciated at birth	Not conclusive	Singh et al. (68)
**GUT**	*Salmonella typhimurium*	Formation of fibrosis regulated by RORα in ILC3s	Intestinal fibrosis	Lo et al. (71)
		Activation of the IL-12/STAT4/IFN-γ signaling pathway in ILC3s by Runx3	Limitation of the intracellular bacterial infection	Yin et al. (72)
	*Giardia Lamblia*	Secretion of IL-17 by ILC3s	Not conclusive	Lee et al. (75)
	*Klebsiella pneumoniae*	Secretion of IL-17, IL-22, and TNF by ILC3s produces LCN-2 by intestinal epithelial cells	Inhibition of *K. pneumoniae* growth *in vitro*	Coorens et al. (77)
		Production of IL-17A by ILC3s	Protection against sepsis by *K. pneumoniae* in newborns	Niu, et al. (78)
	*Yersinia enterocolitica*	Induction of IFNγ by ILC3s induced by HVEM’s ligand LIGHT	Protection	Seo et al. (82)
	*Clostridium difficile*	Regulation of ILC3s by hypoxia	Protection	Fachi et al. (85)
		Production of IL-1β (FFAR2-NLRP3 inflammasome axis) in neutrophils triggered by acetate. Secretion of IL-22 by ILC3s	Protection	Fachi et al. (86)
	*Candida albicans*	Dectin-3 deficiency, IL-7 production, and HIF-1α-dependent glycolysis in macrophages. Induction of IL-22 by ILC3s through STAT3 and AhR	CAC (colitis-associated colon cancer) progression	Zhu et al. (87)
	*Citrobacter rodentium*	Regulation of ILC3s by mTOR	Protection	Blanda Di Luccia et.al. (90)
		Induction of IL-22 via FFAR2 and FFAR3 in ILC3s by butyrate	Protection	W. Yang et al. (91) E. Chun et al. (92), A. Sepahi et al. (93)
		Production of IL-22 in ILC3s modulated by atRA-HIC1 axis	Protection	K. Burrows et al. (94)
		Production of IL-22 in ILC3s modulated by Vitamin D-Cyp27B1 axis	Protection	Y. Lin et al. (95)
		Regulation of ILC3 role and migration by ARNTL (master circadian activator)	Protection	Godinho-Silva et al. (96)
		Inhibition of IL-22 secretion by ILC3s through VIPR2	Susceptibility	J. Talbot et al. (97)
		Regulation of ILC3s by TL1A	Protection	J.Castellanos et al. (98)
		Regulation of ILC3 migration by the expression of GPR183	Migration-Protection	C. Chu et al. (99)
		Secretion of IL-22 by ILC3s triggered by NKR-P1B	Protection	E. Abou-Samra et al. (100)
		Inhibition of IL-17 and IL-22 secretion by ILC3s through RANK-RANKL interaction	Susceptibility	J. Bando et al. (101)
		Regulation of ILC3 maintenance by AhR	Protection	S. Li et al. (104)
		M1 macrophage type induced by GM-CSF-mediated ILC3s	Protection	T. Castro-Dopico et al. (105)
		Pathogen-specific IgA limitation driven by CCR6+ILC3s and TfH cells interaction through MHC-II	Checkpoint: Quality and magnitude control of T-dependent IgA	Melo-Gonzalez et al. (106)

## Author Contributions

AV-N, AO-R and AC-A wrote and prepared the manuscript. MG-S designed the figures. All authors contributed to the article and approved the submitted version.

## Funding

The present review was supported by Ramon y Cajal Program (RYC-2017-21837) and the grant N° RTI2018-093647-B-I00 to AC-A from the Ministerio de Ciencia, Innovación e Universidades (MCIU), Agenda Estatal de Investigación and Fondo Europeo de Desarrollo Regional (FEDER). A.V-N is a recipient of an FPI fellowship (PRE2019-090341) from the Spanish Ministry of Science, Innovation, and Universities.

## Conflict of Interest

The authors declare that the research was conducted in the absence of any commercial or financial relationships that could be construed as a potential conflict of interest.

## Publisher’s Note

All claims expressed in this article are solely those of the authors and do not necessarily represent those of their affiliated organizations, or those of the publisher, the editors and the reviewers. Any product that may be evaluated in this article, or claim that may be made by its manufacturer, is not guaranteed or endorsed by the publisher.
